# A Prediction Model of Human Resources Recruitment Demand Based on Convolutional Collaborative BP Neural Network

**DOI:** 10.1155/2022/3620312

**Published:** 2022-06-24

**Authors:** Haoran Li, Qing Wang, Jiakun Liu, Dawei Zhao

**Affiliations:** ^1^Shandong Youth University of Political Science, Jinan, Shandong 250103, China; ^2^University of International Business and Economics, Beijing 100029, China

## Abstract

This paper presents an in-depth study and analysis of the prediction model of force resource recruitment demand using a convolutional neural network combined with a BP neural network algorithm. BP neural network technology is introduced to be applied to enterprise management talent assessment activities. Using BP neural network has strong parallel processing characteristics, as well as unique adaptive learning and feedback adjustment capabilities while combining the traditional enterprise talent assessment system, to build a business management talent assessment model based on BP neural network technology, to circumvent the possible influence of subjective factors in talent assessment, reduce assessment errors, and improve the accuracy and validity of the assessment. The first layer of convolutional layers may only extract some low-level features such as edges, lines, and corners, and more layers of the network can iteratively extract more complex features from low-level features. The constructed applicant reputation evaluation model based on multiplicative long- and short-term recurrent neural network and the hybrid project recommendation model based on conditional variational self-encoder were experimented on Freelancer's dataset for effectiveness, respectively, and the experimental results showed that the proposed employer hiring decision model, reputation analysis model, and applicant project recommendation model have more reliable performance compared with the existing models. The research results achieve more efficient matching of labor supply and demand in the online labor market and provide technical support for the online labor market platform to realize personalized, intelligent, and accurate services for both employers and applicants.

## 1. Introduction

How to be invincible in the era of the knowledge economy and how to open the winning side in the white-hot competition for talents are the challenges that modern enterprise managers need to think about and must face. Human resource management, as an important grip for modern enterprises to maintain healthy and stable development, plays a decisive role for enterprises to win competition and gain advantages. With talents, enterprises will have the basis to promote the efficient integration and rational use of other resources and will have the guarantee to achieve continuous growth and leapfrog development [1]. Obtain a truly useable data set to pave the way for subsequent algorithm training. In this way, the core competitiveness of the enterprise is mainly reflected in the enterprise's scientific selection, reasonable allocation, efficient management of talents, and the comprehensive management ability to help them obtain growth and development space. Talent measurement and talent evaluation are the fusion of quantitative and qualitative, together as two important means of talent assessment. The predecessor of talent measurement is the psychological test, which mainly focuses on the measurement of human ability, quality, personality, and other personal characteristics, and often uses methods such as situational simulation, psychological test, interview, etc., while talent evaluation is more qualitative-based description, analysis, and judgment, by comparing the previous measurement results with the enterprise performance goals, development vision, and the specific requirements of the relevant positions on the ability and quality of the analysis [2]. The evaluation is based on scientific and reasonable evaluation, thus helping enterprises to open and optimize all aspects of human resource management and development and truly achieve the requirements of “good appointment.”

HR must face a large amount of unstructured data every day, such as all kinds of resumes, recruitment needs of employers, feedback reports of interviews, etc. How to utilize a large amount of information efficiently and fully has become the main battlefield of the human resources department of each company. With the deepening of enterprise information management, using information technology to improve the efficiency of enterprise operation and management is the consensus of contemporary enterprises; however, the lack of accurate enterprise market positioning and the lack of effective demand for talents are also a problem for enterprise HR recruitment [3]. The data collection method in this paper will be divided into source types according to the data collection channels. In recent years, due to the rapid development of the Internet and information technology, it has reduced many HR repetitive process works, such as ATS (Automatic Candidate Management System) which greatly accelerates the interview process and facilitates the management of candidates. Another example is the payroll management system, which makes the management of employees' salaries, social security, etc. simple and clear, but these mainly process automation [4]. However, nonprocess tasks such as screening candidates have not become easier and still require recruiters to spend a lot of time searching and human judgment on the degree of compliance between resumes and job openings.

The existing network recruitment system has a greater functional enrichment than in the past and brings a more convenient recruitment environment to the whole recruitment market. However, there are still some shortcomings, asymmetric access to information for companies and job seekers, nontransparent news between companies and job seekers in the job market, all the information on the job site page being more scattered, and the lack of data analysis of the entire recruitment market data to display the function of the general environment. Secondly, job seekers cannot quantify the specific level of the company, the company also has no intuitive positioning of various aspects of the conditions of job seekers, and job seekers cannot clearly understand their strengths and weaknesses. Finally, the existing job sites are more concerned about the process of active job search for job delivery, and less attention is paid to job seekers for active and personalized job recommendation functions. This thesis aims to crawl the real data through the crawler, excavate the recruitment market supply and demand, design and create portraits for job seekers, help enterprises to accurately search for job seekers and obtain feedback information on job seekers' needs, realize the mapping of job competency requirements in the form of radar diagram for each position, and in addition use the BiLSTM model and introduce the semantic matching model of attention mechanism automatically. The main server uses the memory processing method to process the multisegment data streams in parallel, classify and process the data according to the data source type, and persist it as the original data set. In addition, we use the BiLSTM model and introduce the attention mechanism of the semantic matching model to filter the job information, with deep understanding of the user's conditions and job requirements, and push the most suitable job automatically, quickly, and accurately to the user.

## 2. Related Work

With the development of the recruitment industry, increased companies start to post job information on job boards, and job seekers will browse and find companies that meet their satisfaction on the website. However, there are hundreds of electronic resumes and job postings on job boards every day, and it takes a lot of manpower and time to filter the resumes that meet the company's requirements from the huge number of resumes. The idea of collaborative filtering is that we need to recommend products to users based on their past preferences, combined with the preferences of similar users [5]. The collaborative filtering algorithm requires knowledge of a large amount of historical user data, and if it is a new system, then there is the problem of a cold start of data. CASPER system is the first system that uses collaborative filtering for user recommendation. Content-based recommendation mainly analyzes the items that users liked in the past and finds similar items before recommending them [6]. After the nonlinear processing of the connection function, this layer transmits the processed information to the output layer of the neural network to output the result. There is still the problem of cold start of data and if the user has not used the system, the system is unable to analyze the items that the user likes. Analyze the content of job requirements and job seekers' resumes, and recommend resumes by calculating the similarity between them [7]. A resume recommendation system is envisioned to calculate the similarity between the two based on the textual content of the resume including its career skills and work experience to match the corresponding content in the job requirements. A machine learning algorithm is proposed to extract the semantic features of resumes and job postings to calculate the similarity between resumes and job requirements [8]. The features of resumes and recruitment information are extracted using semantic analysis [9]. The text, the resume, and the recruitment requirements are vectorized, and the similarity between the two vectors is calculated to obtain the matching degree of the resume and the recruitment requirements of the company.

Recommendation algorithms have been widely used in e-commerce, music, and movies in recent years, and they have been combined with popular technologies to iterate and update the recommendation algorithms with good results [10]. However, in the field of human resources, the recommendation algorithm mainly adopts the traditional collaborative filtering and content-based recommendation method, which still requires users to spend more time and energy to discern whether the job information is suitable for them, and there are problems such as cold start and data sparsity, making human resources recommendations cannot get good results. With the strengthening of the network of employment resources, the recommendation algorithm has been developed accordingly [11]. The research direction in the field of HR recommendation also improves the traditional recommendation algorithm, improves the traditional employment recommendation method, proposes the calculation strategy of combining interest sensitivity, uses the recruitment data of different enterprises for job seekers to calculate the interest sensitivity of enterprises-users, and uses it to improve the similarity calculation among job seekers [12].

Most of the algorithms currently applied to HR recommendation are mature and stable traditional recommendation algorithms such as collaborative filtering recommendation algorithms or improved algorithms that innovate on the traditional algorithms. The data collection adopts the dynamic and direct processing mode, which is different from the traditional mode of first storing and then processing, which does not need to complete the data accumulation and landing first. The algorithms are implemented singly and do not make full use of the advantages of emerging technologies, which are not ideal in terms of performance and recommendation personalization intensity. In this paper, we hope to further research and improve the algorithm with the help of emerging technologies such as deep learning and improve the quality of human resource recommendation with the high efficiency and stronger representation ability to emerging technologies to alleviate the current employment problem of “job mismatch,” so the algorithm research in this paper has certain practical significance. Therefore, based on a large amount of real data available in the online labor market, this paper explores and researches the employer recruitment decision problem, applicant reputation evaluation problem, and applicant project recommendation problem on the online labor market platform by using a data-driven approach and deep learning to provide reliable technical support to realize personalized, intelligent, and accurate services to meet the higher demand of online labor services.

## 3. Analysis of the Convolutional Cooperative BP Neural Network HR Recruitment Demand Prediction Model

### 3.1. Convolutional Collaborative BP Neural Network Algorithm Design

The human brain is the most efficient and complex information processing system known to us up to now. People have tried to explore the working principle of the brain nervous system by conducting a lot of research in anatomy, physiology, neurology, brain science, psychology, cognition, and other disciplines [13]. It is based on these studies that humankind has accomplished the great invention of replacing part of the functions of the human brain with machines or computers, which help the human brain to perform functions such as memory, arithmetic, and judgment. However, because the electronic components that make up a computer cannot reach the reflection speed of nerve cells, they cannot effectively handle complex problems such as image recognition, associative reasoning, and language comprehension. The artificial neural network has strict structure, strong operability, and stable operating conditions. Because of its strong self-adaptation and high nonlinearity, it can be executed without establishing a special mathematical model when dealing with complex problems, nonlinear relationship information.

**Figure 1:**
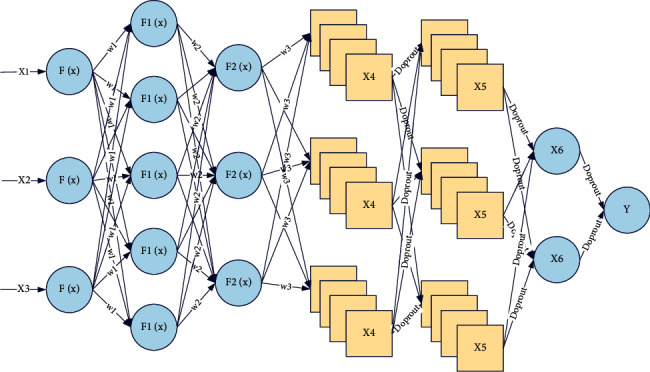
Convolutional cooperative BP neural network framework.

Artificial neural network is a data processing discipline that has emerged only in recent years. Based on the in-depth study of the brain nervous system and neuron structure, the artificial neural network has been created utilizing simulation and emulation, which consists of many interrelated multiple artificial perceptual machines. The main functions of the artificial perceptual machines described here include determining the combination of input signals, output signals, and the corresponding weights [14]. Artificial neural networks have a rigorous structure, strong operability, and stable operating conditions, due to their strong adaptiveness and high nonlinearity, thus allowing the execution of nonlinear relational information without the need for specialized mathematical models when dealing with complex problems. Therefore, artificial neural networks are widely used in many fields such as data compression, pattern recognition, and classification training with their excellent features, which is shown in Figure 1.

It is a technical tool with distributed parallel processing capability as well as the ability to effectively process nonlinear problems with ambiguous information in complex environments utilizing adaptive learning and feedback adjustment. The basic principle is to implement the self-learning process in two steps. In the first step, the signal is propagated forward from the input layer through the connection nodes to the implicit layer, and after the connection function is processed nonlinearly, the processed information is then passed from this layer to the output layer of the neural network to output the result.

(1) $$\begin{array}{c} h_{k}^{2}=\sum_{i=1}^{m}w_{ki}^{2}x_{i}^{2}+\theta_{k}^{2}. \end{array}$$

Meanwhile, by training a large amount of sample data, the laws can be summarized appropriately in the output layer to improve learning efficiency. If the output value of the forward propagation in the first stage does not match the expected value, the training process will automatically shift to the second step, which is the reverse transmission of the error signal. You can see that the accuracy is 0.679, the precision is 0.682, the recall is 0.646, the F1 is 0.663, and the AUC is 0.736. And compare the APJFNN model with one of the popular machine learning algorithms that perform well in the classification field. In this stage, the error between the actual output value and the expected value is obtained by arithmetic, and the error is transmitted in the opposite direction of the original path, and the error value is assigned to all neural nodes in each layer.

(2) $$\begin{array}{c} z_{k}=f\left(\sum_{i=1}^{m}w_{ki}^{2}x_{i}^{2}+\theta_{k}^{2}\right). \end{array}$$

The connection weights and thresholds are also adjusted simultaneously according to the error gradient descent method. To achieve the effect of error convergence, the actual output of the BP neural network will be as close to the expected value as possible [15]. The main functions of the artificial perceptron mentioned here include determining the combination of input signals, output signals, and corresponding weights. The above two processes are carried out alternately, and the connection weights are continuously adjusted until the error converges to the present value or the expected learning effect is achieved, which is the training and learning process of BPNN. After learning and training, the BP neural network also needs to be tested for accuracy and validity by test samples to verify the precision and accuracy of the network calculation.

(3) $$\begin{array}{c} o_{j}=\sum_{k=1}^{p}\mu_{jk}\cdot f\left(\sum_{i=1}^{m}w_{ki}^{2}x_{i}^{2}+\theta_{k}^{2}\right)+\varphi_{j}. \end{array}$$

Consider a two-dimensional (2D) image input with neuron channel *c*=3, for example, RGB color values. Define a single channel of the first layer of the convolution kernel as the input image *r* × *r* region (called the receptive domain of the convolution kernel) for this convolution kernel and compute the weighted sum. This convolution kernel can be applied at every possible offset in the input image. If there are c' such channels, the parameters of the layer can be expressed as a tensor K of size *c*′ × *c* × *r* × *r* and the response of the channel at any offset (*a*, *b*) is as follows:

(4) $$\begin{array}{c} O\left(a,b\right)=\sum_{u=1}^{r_{1}}\sum_{v=1}^{r_{2}}\left(K\left[u,v\right]\right)⊙I\left[a+u,b+v\right]. \end{array}$$

The recommendation algorithm based on user filtering focuses on finding a group of users like the user who is ready to be recommended and then recommending to it items from that group that is not recommended to that user. The recommended method is a clustering process, where users with the same interests are gathered. First, find the set of users who are like the recommended user. Similarly, for resumes that fail to predict, the model cannot explain where the resume is poorly written.

(5) $$\begin{array}{c} \text{Sim}\left(u,v\right)=\frac{N\left(u\right)\cup N\left(v\right)}{N\left(u\right)\cap N\left(v\right)}. \end{array}$$

The input data is only linearly mapped at the convolutional layer, and the representational power of a neural network with only convolutional operations is limited [16]. The nonlinear activation layer exists as the name implies to increase the nonlinear representation of the neural network. The nonlinear characterization ability of the nonlinear activation layer comes from the specific nonlinear activation function, and the common nonlinear activation functions are Sigmoid functions.

Firstly, all the input information eventually needs to be set to a specified length, and using a fixed-length encoding to represent the information would cause the longer input information to be partially lost. However, it is difficult to do this with classical encoding-decoding structures.

(6) $$\begin{array}{c} c_{j}=\sum_{i=1}^{T}\alpha_{ij}^{2}h_{j}^{2}. \end{array}$$

**Figure 2:**
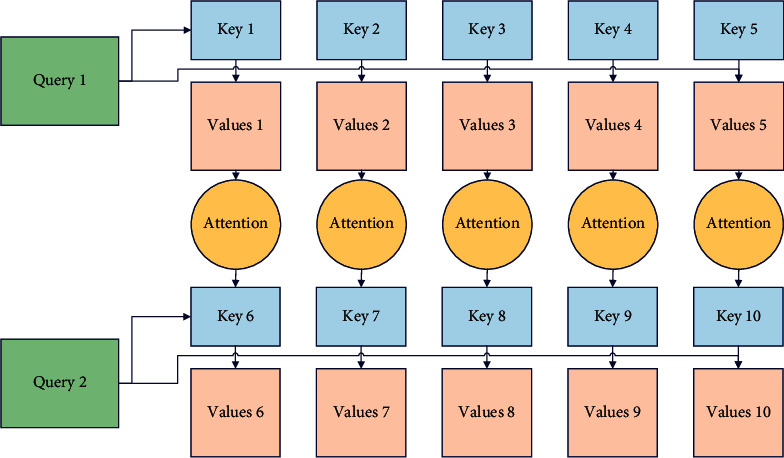
Substance of the attention mechanism.

As shown in Figure 2, this feature significantly reduces the number of parameters of the convolutional neural network and significantly increases the size of the network without increasing the training data accordingly. Even so, the model still contains many parameters, so there is a possibility of overfitting; i.e., the model performs well on the training data but not on the independent test set. To improve the generalization ability of the deep selection model and prevent the overfitting problem, a common solution strategy is the regularization method. Regularization methods are a class of strategies that are shown to be designed to reduce testing errors at the cost of potentially increasing training errors. In actual scenarios, the interpretability of the model is still relatively weak. In the context of deep learning, most regularization strategies regularize the estimates. The regularization of estimates trades an increase in bias for a decreased invariance.

(7) $$\begin{array}{c} P\left(b_{i}^{k}|X^{k}\right)=\frac{\exp\left(U_{i}^{k}\right)}{\sum_{j=1}^{m}\exp\left(U_{i}^{k}\right)}. \end{array}$$

A computationally convenient but powerful class of regularization methods, Dropout, was chosen. The integration of Dropout training includes all subnetworks formed after removing nonoutput units from the base network. During the training of the model, Dropout randomly reviews (or zeroes) the response of each neuron with probability 1-p. Intuitively, this prevents the model from relying too much on the response value of any neuron, thus reducing redundancy in the learning representation [17]. Dropout is more efficient than another standard computationally inexpensive regularization method (e.g., weight decay, filter parametric constraints, and regularization of sparse activations) and performs well on models that can all be trained with stochastic gradient descent.

### 3.2. HR Recruitment Demand Prediction Model Design

In the overall process of the recommendation algorithm in this paper, data collection is the primary prerequisite, and its main task is to collect the source data for the core model training of the algorithm and persist it as a dataset to be used for subsequent data preprocessing and core model training of the algorithm. The HR domain dataset used in this paper comes from a comprehensive social security business service project undertaken by our lab team [18]. To improve the generalization ability of the depth selection model and prevent the overfitting problem, the common solution is the regularization method. With the help of the business service of the social security business company and the online operation of the project, the initial raw dataset of human resources is acquired by collecting the relevant job search information of the participants.

After the collection task, the initial raw data needs to be preprocessed with rules based on the relevant characteristics of the human resource domain, which is used to normalize the collected data and get the real useable data set for the subsequent algorithm training. The data collection method in this paper will be divided into source types according to the data collection channels, which is useful for both the construction of recommendation datasets and the related data analysis and information statistics for different channel sources, to facilitate the realization of visual analysis requirements.

**Figure 3:**
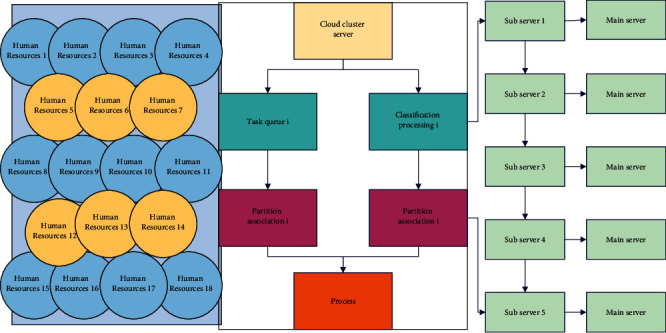
Data collection partition task queue construction and data categorization design diagram.

In the face of massive data, data collection is particularly important. For many resume providers, they need to know not only whether they match, but also how to match and where they do not match, so that they can improve their business in the future. If quality data collection cannot be carried out effectively, it will affect the training of the model and cannot generate a model with strong enough recommendation performance for accurate job information recommendation. The information acquisition between companies and job seekers is asymmetric, the information between companies and job seekers in the job market is opaque, all information on the recruitment website page is relatively scattered, and there is a lack of data analysis on the general environment data of the entire recruitment market. The current existing data collection solutions provide a parallel multithreaded approach for data collection, but generally, only business data collection of the same business type is performed on a single machine. Due to the traditional business data sources being less, the information entry can only be done through the Social Security Bureau in the past, and the data level is light, and the single-machine data collection can meet the collection needs. However, in the era of big data development, the entry of relevant social security information is no longer limited to the on-site entry method of the Social Security Bureau but also extended to the entry of social security card terminal devices and mobile applications. The diverse collection levels of massive data make the standalone collection server unable to support the collection of large data stably and efficiently, and there is a large performance bottleneck, as shown in Figure 3.

**Figure 4:**
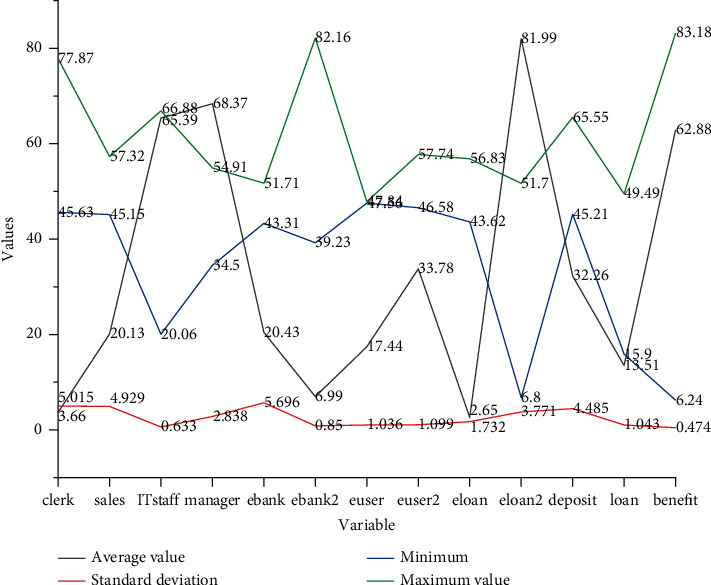
Descriptive statistics of variables.

One of the research objects of concern in this paper is the number of bank employees, especially considering that the digital transformation has positive and negative effects on the number of employees in different sequences, according to the analysis above, this paper conducts regression analysis on the personnel in counter sequence, marketing sequence, technology sequence, and management sequence, which are more affected by the digital transformation, and the explanatory variables are chosen to measure the number of employees in these four positions, respectively, to measure the human resources. The change in the number of employees is shown in Figure 4.

Figure 4 shows that the correlation coefficients among some explanatory variables are above 0.8, for example, among deposits, loans, profits, total wages, average daily counter business volume, number of individual customers, and number of business outlets, indicating that there are high linear correlations among these variables, and if these variables are included in the regression model, the model will have serious multicollinearity and lose credibility or have difficulty in estimating the parameters accurately. However, when it tends to be stable, the effect is not as good, which means that when the learning rate is greater than 0.3, the model training becomes difficult and is not the optimal state of the model.

The module mainly includes user registration, a user login, and account management, and for business users, it is mainly business registration, login, and management of business accounts. For job seeker users, it is mainly for job seeker registration, login, and job seeker account management [20]. First, choose personal registration or enterprise registration by identity, enter the registration interface, personal registration enter e-mail and password, repeat password and name, then enter the login interface, for enterprise registration, enter e-mail, password, repeat password, enterprise name, financing stage, industry sector, and company profile, and then enter the login interface, and enter e-mail and password to log in. After that, you can enter the account management section to add, delete, and modify the account information.

## 4. Analysis of Algorithm Performance Results

**Figure 5:**
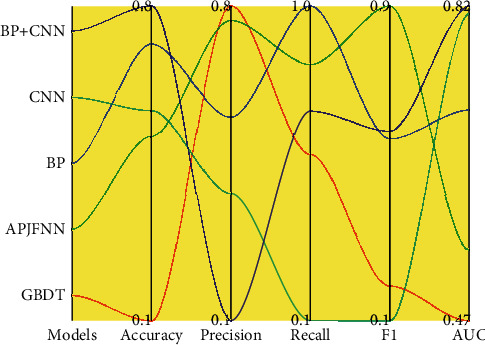
Algorithm results metrics.

The test data consists of *n* sets of exposed job data; each set of data contains a job seeker and a sequence of exposed job candidates. Each set of jobs needs to be predicted and sorted to give the sorted job sequence. To fully validate the performance, we choose the AUC metric to measure the performance. In addition, accuracy, precision, recall, and F1 are also used as evaluation metrics. The accuracy is 0.679, precision is 0.682, recall is 0.646, F1 is 0.663, and AUC is 0.736. The results of the APJFNN model are compared with those of GBDT, one of the popular machine learning algorithms that perform well in the classification field, as shown in Figure 5.

This model makes full use of the ability of the attention mechanism and sets 4 places of attention, which makes the learning ability of the model very powerful. It considers the different effects of the same work experience on different job requirements, which is equivalent to “dynamic keyword extraction” and makes the model have a certain interpretation. At the same time, it also brings a more convenient recruitment environment to the entire recruitment market. However, there are still some deficiencies. However, for the predicted successful resumes, for example, two resumes with scores of 0.6 and 0.8, respectively, the model cannot explain why the resume with a score of 0.8 is better than the one with a score of 0.6. Similarly, for a resume that fails, the model cannot explain what is wrong with the resume. In a real-world scenario, the explanations of the model are still relatively weak, and many resume providers need to know not only whether they match, but also how and where they do not match so that they can improve their business in the future.

**Figure 6:**
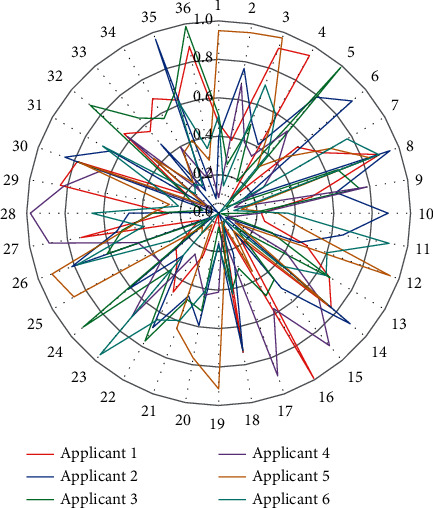
Typical example of employer hiring decision.

Five applicants applied for the project, and each applicant's attributes are displayed in a polar plot, with each colored line representing the 25 attributes of the applicant who applied for the project and the radius indicating the normalized value of the corresponding attribute. For each attribute, the applicant's score is scaled in the range [0, 1] based on the maximum and minimum values of the five applicants. Regularization methods are a class of strategies explicitly designed to reduce test error at the cost of potentially increasing training error. Hired applicants are indicated by a thick red line and other applicants are indicated by thin lines of other colors. Applicant 1 shows strengths in terms of professionalism, quality of work, degree of return to employment, skill level, communication level, and overall evaluation that can explain to some extent his success in hiring, as shown in Figure 6.

**Figure 7:**
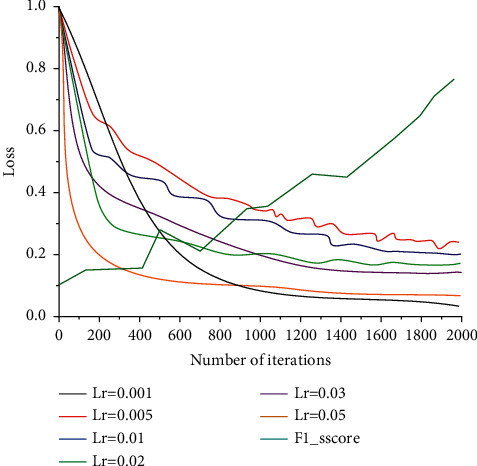
Loss comparison with different learning rate parameters.

Figure 6 compares the experimental results of the conditional logic model and the selected depth model in the training set, validation set, and test set in the twelve categories of the project. The selected deep selection model structure and hyperparameters are configured with four convolutional layers, a convolutional channel size of 100 per layer, a learning rate of 0.01 for SGD, and a Dropout rate of 0.3. *L* and ACCACC2 and ACC are used to evaluate the effectiveness of the model on the training set, validation set, and test set. From the four-evaluation metrics, the deep selection model showed better prediction accuracy in almost all types of items compared to the conditional logic model. On average, the standardized log-likelihood L-value of the deep selection model improved by 0.090–0.253, Moreover, according to the F1-Score comparison results in Figure 7, the F1-Score obtained when the learning rate is 0.3 is the best, while the ACCL ACC and ACC metrics improved by 0.015–0.041.

In this paper, a multichannel convolutional submodel is designed to optimize the unique feature representation of a single convolution, which is used to learn multiple holistic feature representations by using different convolutional filters to traverse the local data blocks, constituting a more complex feature mapping representation. However, different numbers of convolutional channels have an impact on the overall model performance. In this section, we will experimentally compare different numbers of convolutional channels to select the appropriate number of convolutional channels to determine the overall model structure.

## 5. Experimental Results of Demand Forecasting Model Application

**Figure 8:**
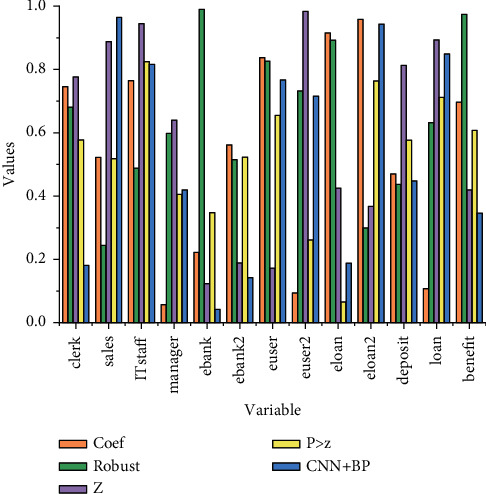
Parameter estimation results of the people model.

The estimated coefficient for online personal customers is significantly positive, which is consistent with the possible results of the analysis in the previous section of this paper. Each additional 10,000 online personal customers increase the number of marketing sequence personnel by approximately 0.29. At this place, we need to analyze and discuss in depth the impact of online individual customers on the human resource requirements of the marketing sequence. The mixed pooling strategy is used to improve the loss of feature information in the max pooling process; the problem of insufficient learning of hard-to-classify samples is solved by using the improved cross-entropy loss function. Because, theoretically, with the increase of online individual customers, the human resource demand of marketing sequence should be decreased, but the model estimated coefficient results show positive. We analyze it mainly because of two reasons: first, although the workload of traditional offline business front-end such as information filling forms and customer information system entry of commercial banks decreases with the increase of online individual customers, the workload of back-end postloan service and postloan customer management is still there due to the current development stage, and this part of the workload is still increasing at the same time with the increase of customer scale, and there is still a certain demand for marketing sequence personnel. On the other hand, although the traditional offline workload has been reduced, functions and requirements such as customer marketing have been strengthened, increasing the demand for human resources in the marketing sequence. We expect that, with the advancement of digital transformation and the enhancement of the intelligence level of the customer postloan management system, the demand for marketing sequence personnel for back-end postloan services and postloan customer management will be further reduced in the future, continuing to release the marketing sequence personnel workforce. Therefore, we believe that the positive effect of online individual customers on the demand for marketing sequence human resources is likely to be phased, and the reverse effect is likely to occur in the future as the digital transformation advances, as shown in Figure 8.

The experimental results are shown in Figure 7, and the F1-Score comparison results obtained from the experiments are shown in Figure 7.

The model stabilizes at about 100 iterations, and the model reaches the basic convergence state. Talent measurement and talent evaluation are the integration of quantitative and qualitative, and together they are two important means of talent evaluation. The overall loss of the model is minimized when the learning rate is set to 0.3, and the model is in the best condition. When the learning rate is less than 0.3, the overall loss value is large, and the loss value decreases as the learning rate increases, which indicates that the learning rate less than 0.3 does not make the model optimal. It becomes difficult and is not optimal for the model. Moreover, the F1-Score comparison in Figure 7 shows that the best F1-Score is achieved at a learning rate of 0.3. Therefore, the learning rate is set to 0.3 in this paper, and the subsequent experiments will be conducted at this learning rate.

## 6. Conclusion

Gradient boosting tree and convolutional neural network technology are combined to implement a recommendation algorithm applied to human resources field, using the feature transformation ability of gradient boosting tree and the high-level feature learning ability of a convolutional operation to optimize the recommendation performance, solving the problem of limited model feature extraction ability of traditional recommendation algorithm, and improving the recommendation quality. However, the lack of precise enterprise market positioning and the lack of effective manifestation of the demand for talents are also a difficult problem in enterprise HR recruitment. To address the shortcomings of the training process of the hybrid convolutional neural network, the activation function, pooling strategy, and training loss function are optimized, and the feasibility of the optimization strategy is verified by comparison experiments, which can effectively improve the quality of human resource recommendation. In the field of HR recommendation, the interest degree of job seekers' job intention changes with time. The current recommendation algorithm in this paper does not consider the temporal factor, so in the subsequent improvement, we will try to add the temporal factor and use the recurrent neural network RNN model to deal with the temporal factor and optimize the recommendation algorithm.

## Data Availability

The data used to support the findings of this study are available from the corresponding author upon request.

## References

[1] Dalal S., Khalaf O. I. (2021). Prediction of occupation stress by implementing convolutional neural network techniques. *Journal of Cases on Information Technology*.

[2] Liu M., Jervis M., Li W. (2020). Seismic facies classification using supervised convolutional neural networks and semisupervised generative adversarial networks. *Geophysics*.

[3] Khalil R. A., Saeed N., Masood M., Fard Y. M., Alouini M. S., Naffouri T. Y. A. (2021). Deep learning in the industrial internet of things: potentials, challenges, and emerging applications. *IEEE Internet of Things Journal*.

[4] Ji Y., Zhang J., Wang X., Yu H. (2018). Towards converged, collaborative and co-automatic (3C) optical networks. *Science China Information Sciences*.

[5] Buhrmester V., Münch D., Arens M. (2021). Analysis of explainers of black box deep neural networks for computer vision: a survey. *Machine Learning and Knowledge Extraction*.

[6] Anitha S., Vanitha M. (2021). Optimal artificial neural network-based data mining technique for stress prediction in working employees. *Soft Computing*.

[7] Huynh E., Hosny A., Guthier C., Aerts H. J. W. L, Mak R. H (2020). Artificial intelligence in radiation oncology. *Nature Reviews Clinical Oncology*.

[8] Chiu M. C., Chuang K. H. (2021). Applying transfer learning to achieve precision marketing in an omni-channel system–a case study of a sharing kitchen platform. *International Journal of Production Research*.

[9] Wang F., Preininger A. (2019). AI in health: state of the art, challenges, and future directions[J]. *Yearbook of medical informatics*.

[10] Lima A. L. C. D., Aranha V. M., Carvalho C. J. L., Nascimento E. G. S. (2021). Smart predictive maintenance for high-performance computing systems: a literature review. *The Journal of Supercomputing*.

[11] Meng L., McWilliams B., Jarosinski W. (2020). Machine learning in additive manufacturing: a review. *Journal of Occupational Medicine*.

[12] Bublitz E., Nielsen K., Noseleit F., Timmermans B. (2018). Entrepreneurship, human capital, and labor demand: a story of signaling and matching. *Industrial and Corporate Change*.

[13] Shashidhara S., Mitchell D. J., Erez Y., Duncan J. (2019). Progressive recruitment of the frontoparietal multiple-demand system with increased task complexity, time pressure, and reward. *Journal of Cognitive Neuroscience*.

[14] Rispel L. C., Blaauw D., Ditlopo P., White J. (2018). Human resources for health and universal health coverage: progress, complexities and contestations. *South African Health Review*.

[15] Kulkarni S. B., Che X. (2019). Intelligent software tools for recruiting. *Journal of International Technology and Information Management*.

[16] Correll C. K., Ditmyer M. M., Mehta J. (2015). American college of rheumatology workforce study and demand projections of pediatric rheumatology workforce, 2015–2030. *Arthritis Care & Research*.

[17] Huang D., He H., Liu T. (2021). City size and employment dynamics in China: evidence from recruitment website data. *Journal of Geographical Sciences*.

[18] Vu T. L. A., Le T. Q. (2019). Development orientation for higher education training programme of mechanical engineering in industrial revolution 4. 0: a perspective in vietnam. *J. Mech. Eng. Res. Dev*.

[19] Zeebaree S. R. M., Shukur H. M., Hussan B. K. (2019). Human resource management systems for enterprise organizations: a review. *Periodicals of Engineering and Natural Sciences*.

[20] Sindelar P. T., Pua D. J., Fisher T., Peyton D. J, Brownell M. T., Williams L. M. (2018). The demand for special education teachers in rural schools revisited: an update on progress. *Rural Special Education Quarterly*.

